# Immune Reactions against Gene Gun Vaccines Are Differentially Modulated by Distinct Dendritic Cell Subsets in the Skin

**DOI:** 10.1371/journal.pone.0128722

**Published:** 2015-06-01

**Authors:** Corinna Stefanie Weber, Katrina Hainz, Tekalign Deressa, Helen Strandt, Douglas Florindo Pinheiro, Roberta Mittermair, Jennifer Pizarro Pesado, Josef Thalhamer, Peter Hammerl, Angelika Stoecklinger

**Affiliations:** 1 Department of Molecular Biology, University of Salzburg, Salzburg, Austria; 2 Central Animal Laboratories, University of Salzburg, Salzburg, Austria; 3 Christian Doppler Laboratory for Allergy Diagnosis and Therapy, University of Salzburg, Salzburg, Austria; INSERM, FRANCE

## Abstract

The skin accommodates multiple dendritic cell (DC) subsets with remarkable functional diversity. Immune reactions are initiated and modulated by the triggering of DC by pathogen-associated or endogenous danger signals. In contrast to these processes, the influence of intrinsic features of protein antigens on the strength and type of immune responses is much less understood. Therefore, we investigated the involvement of distinct DC subsets in immune reactions against two structurally different model antigens, *E*. *coli* beta-galactosidase (betaGal) and chicken ovalbumin (OVA) under otherwise identical conditions. After epicutaneous administration of the respective DNA vaccines with a gene gun, wild type mice induced robust immune responses against both antigens. However, ablation of langerin^+^ DC almost abolished IgG1 and cytotoxic T lymphocytes against betaGal but enhanced T cell and antibody responses against OVA. We identified epidermal Langerhans cells (LC) as the subset responsible for the suppression of anti-OVA reactions and found regulatory T cells critically involved in this process. In contrast, reactions against betaGal were not affected by the selective elimination of LC, indicating that this antigen required a different langerin^+^ DC subset. The opposing findings obtained with OVA and betaGal vaccines were not due to immune-modulating activities of either the plasmid DNA or the antigen gene products, nor did the differential cellular localization, size or dose of the two proteins account for the opposite effects. Thus, skin-borne protein antigens may be differentially handled by distinct DC subsets, and, in this way, intrinsic features of the antigen can participate in immune modulation.

## Introduction

Dendritic cells (DC) in the skin comprise at least five subpopulations with different ontogenies and phenotypes [1–4]. Epidermal Langerhans cells (LC) and two dermal subsets express the C-type lectin langerin/CD207 [5–7]. In addition, the dermis accommodates two langerin^neg^ DC subsets that are positive or negative for CD11b, respectively [8]. In face of this complexity it was for a long time difficult to dissect the roles of individual DC subsets in vivo. Transgenic mouse models for constitutive or inducible LC deficiency [9, 10], or diphtheria toxin (DT)-mediated ablation of all langerin^+^ subsets [11, 12] have now greatly assisted in the investigation of skin DC functions in vivo. For some immune reactions, e.g. contact hypersensitivity (CHS) to haptens, skin DC appeared functionally redundant [13]. In other systems, distinct DC subsets displayed a clear functional specialization, with differential roles in CTL activation, Th cell polarization, antigen cross presentation, the induction of tolerance, or the maintenance of homeostasis in the commensal skin flora [8, 14–17].

Functional diversity among skin DC was also observed with skin-borne neo-antigens. In previous studies we found that langerin^+^ dermal DC (LdDC) but not epidermal LC were required for cytotoxic T lymphocytes (CTL) and IgG1 antibodies against beta-galactosidase (βGal) after gene gun (GG) vaccination [18, 19]. Gene gun vaccination involves the transfection of host cells by DNA-coated gold particles that are propelled onto the skin surface by a pulse of pressurized gas. Hence, the antigen itself is not part of the vaccine but, rather, produced by host cells. Importantly, with this method both, the substance administered (plasmid DNA), as well as the vaccination procedure is always the same, irrespective of the antigen. Therefore, we hypothesized that under these conditions any differences in immune reactions originate from intrinsic features of the expressed antigens.

It is well known that intrinsic features of antigens can profoundly influence the strength and type of an immune response. These include parameters such as structural stability, protease activity and others [20–22]. However, with only few exceptions the underlying mechanisms are barely understood. We hypothesized that neo-antigens originating from skin cells could induce different types of immunity by being differentially handled by distinct skin DC subsets. To investigate this, we compared GG vaccines encoding βGal or chicken ovalbumin (OVA) in wild type mice or mice deficient in, either, all langerin^+^ DC or only epidermal LC. Using this model, we found that the two antigens were handled by distinct skin DC subsets in fundamentally different ways.

## Material and Methods

### Mice

C57BL/6N wild type (WT) mice were obtained from Charles River (Germany) or Janvier (France). Langerin-DTR-EGFP (langDTR) [12] mice were backcrossed onto C57Bl/6 for at least 6 generations at the local animal facility. langDTR and OT-I (Ly5.1) transgenic mice were maintained at the local animal facility under specific pathogen-free conditions [23]. Stable groups of 4 to 6 females per cage were used between 8 to 10 weeks (wild types) or 6 to 16 weeks of age (transgenics) at the start of experiments. Animals were kept in individually ventilated cages (Type II long, Tecniplast, Germany) at 65 air changes per hour maintaining positive cage pressure, with access to sterilized chow and autoclaved water *ad libitum*. Fresh cages with woodchip bedding and paper-based nesting material, all autoclaved, were provided weekly. Animal rooms were kept at 20–22°C and 45–65% relative humidity with automated light/dark periods of 12/12 hrs. Animals are daily inspected for any signs of discomfort.

### Ethics Statement

Animal experiments were conducted in accordance with EU guidelines 86/609/EWG and national legal regulations (TVG 2012) and all efforts were made to minimize or avoid suffering. Experiments were approved by the Austrian Ministry of Science, Ref. II/3b (Gentechnik und Tierversuche), permission no GZ 66.012/0014-II/3b/2011. For terminal analysis, mice were euthanized by cervical dislocation.

### Ablation of langerin^+^ DC

For continuous depletion of all langerin^+^ DC, 1 μg DT (Sigma-Aldrich) in 100 μl pyrogen-free PBS was injected i.p. 1d before GG immunization, followed by 0.4μg DT at 3d intervals for the entire duration of experiments. For selective ablation of epidermal LC, mice received a single dose of 1 μg diphtheria toxin 1 wk before GG immunization.

### DNA vaccines and Gene gun Immunization

Gene gun (GG) vaccine plasmid constructs were all based on the eukaryotic expression vector pCI (Promega, Madison, WI). pCI-βGal and pCI-OVA have been described [19, 24]. pCI-cherryOVA was generated by fusing the coding sequence of OVA to the C-terminus of mCherry [25]. pCI-βGalOVA fusion plasmid was generated by PCR techniques, abutting the open reading frame (ORF) for the C-terminal protein right behind the last codon of the N-terminal fusion partner of which the stop codon had been deleted. K14-vaccines were generated by substituting a genomic 2.9 kb fragment just 5’ to the start codon of mouse keratin-14 for the CMV promoter between the unique BglII/PstI sites of the pCI vector. GG immunization was performed either one day after DT treatment when mice were depleted of all langerin^+^ DC or not before 1 wk post DT treatment, to ensure that LdDC have started to repopulate the skin but LC were still absent. All mice were GG boosted after 1 wk. With each shot, 1 μg of plasmid DNA, immobilized onto 0.5 mg gold particles (1.6μm, BioRad, Richmond CA), was delivered with pressurized helium gas at 400 psi using a Helios gene gun (Bio-Rad, Richmond, CA) onto the shaved abdominal skin by two non-overlapping shots. Potential stress caused by noise from the gene gun shots was minimized by immunizing animals in a remote laboratory.

### Serum Antibodies

Serum was prepared from blood collected, either from the tail vein, or the heart of sacrificed mice. Antigen (Ag)-specific serum IgG1 or IgG2c was detected by ELISA with an isotype-specific peroxidase-conjugated Ab and chromogenic development. Antibody titers were determined by end point titration and expressed as the dilution factor yielding a response equal to the detection limit (i.e., mean + 3 x SD of 16 blank values).

### *In vivo* proliferation assay

Erythrocyte-depleted spleen cells from naive OT-I donor mice (Ly5.1) that are transgenic for a TCR with specificity for H2-K^b^–restricted OVA_257–264_ (SIINFEKL) and naïve C57BL/6N (Ly5.2) were stained with 3 μM CFSE (Molecular Probes) for 10 min at 37°C in PBS, 5% FCS. After washing with PBS twice, OT-1 and WT cells were mixed at a 1:1 ratio and from this mix 4x10^6^ cells in 100 μl PBS were injected i.v. into langDTR or WT mice that had been treated with 1 μg DT. Next day, mice were gene gun immunized with pCI-OVA, and cells were isolated from draining LN at indicated time points. Cells were stained with PE-conjugated anti-Vβ5.1,5.2 TCR mAb (clone MR9-4; BD Pharmingen), APC-conjugated anti-CD8 (clone 53.67), APC-Cy7-conjugated anti-Ly5.1 mAb (clone A20, eBioscience) and proliferation of OT-1 cells was analyzed by flow cytometry. Fold expansion of OT-1 cells was calculated by normalizing to injected WT cells.

### ELISPOT for IFNγ- or IL4-secreting splenocytes

ELISPOTS were performed as described [18]. Briefly, spleen cells were cultured for 24hrs with 10μM CTL peptides or 20 μg/ml protein in 96-well filter-bottom Multiscreen plates (Millipore) coated with cytokine-specific mAbs. Cytokine spots were detected with biotinylated matched-pair mAbs and peroxidase-conjugated streptavidin by chromogenic enzyme reaction. Spots were counted manually from 600 dots per inch flat bed scans of duplicate wells.

### *In vivo* CTL assay

CTL activity was determined in vivo as described [26]. Briefly, syngeneic spleen cells were stained with 5 or 0.3 μM CFSE, respectively. The latter fraction was pulsed with 10 μM CTL peptide and combined with the first fraction. Of this mixture, 4 x 10^6^ cells were injected i.v. into recipient mice. After 16 hrs, splenocytes were analyzed by flow cytometry, and % specific lysis was calculated from the reduction of peptide-pulsed CFSE^dim^ cells relative to non-pulsed CFSE^hi^ reference cells.

### Flow cytometric analysis of skin immune cells

Isolation and analysis of skin lymphocytes [18, 27] were carried out as described. Briefly, after removing fur with an electric groomer, trunk skin was separated from subcutaneous fat tissue, cut to small pieces and, for lymphocyte analysis, digested for 45 min. at 37°C in RPMI 1640 containing collagenase XI (4000 U/ml), hyaluronidase (260 U/ml) and DNase (0.1mg/ml), all from Sigma-Aldrich. Cells were harvested by filtration, washed and stained, first for surface markers with mAbs CD45.2-PerCP, CD3-APC-eFluor780, CD4-FITC, CD25-PE, CTLA4-APC, ICOS-PE-Cy7, and live/dead-eFluor506, then fixed and permeabilized and stained with FoxP3-eFluor450, all from eBioscience. For DC analysis, digestion was with 150μg/ml Liberase and 120μg/ml in HBSS and cells were stained with mAbs CD11c-PerCPCy5.5 and CD103-PE (both BioLegend) and, after fixation and permeabilization, with anti-langerin-Alexa488 (clone 929F3.01, Dendritics).

### Statistical analysis

Results of CTL activity, Ab titers and ELISPOTs are presented as arithmetic means ± SD. Unless indicated otherwise in the figure legends, data were tested for statistical significance by one-way ANOVA with Tukey´s multiple Comparison Test. p-values < 0.05 were considered statistically significant.

## Results

### Depending on the antigen, langerin^+^ DC either enhance or suppress CD8^+^ T cell responses

**Fig 1:**
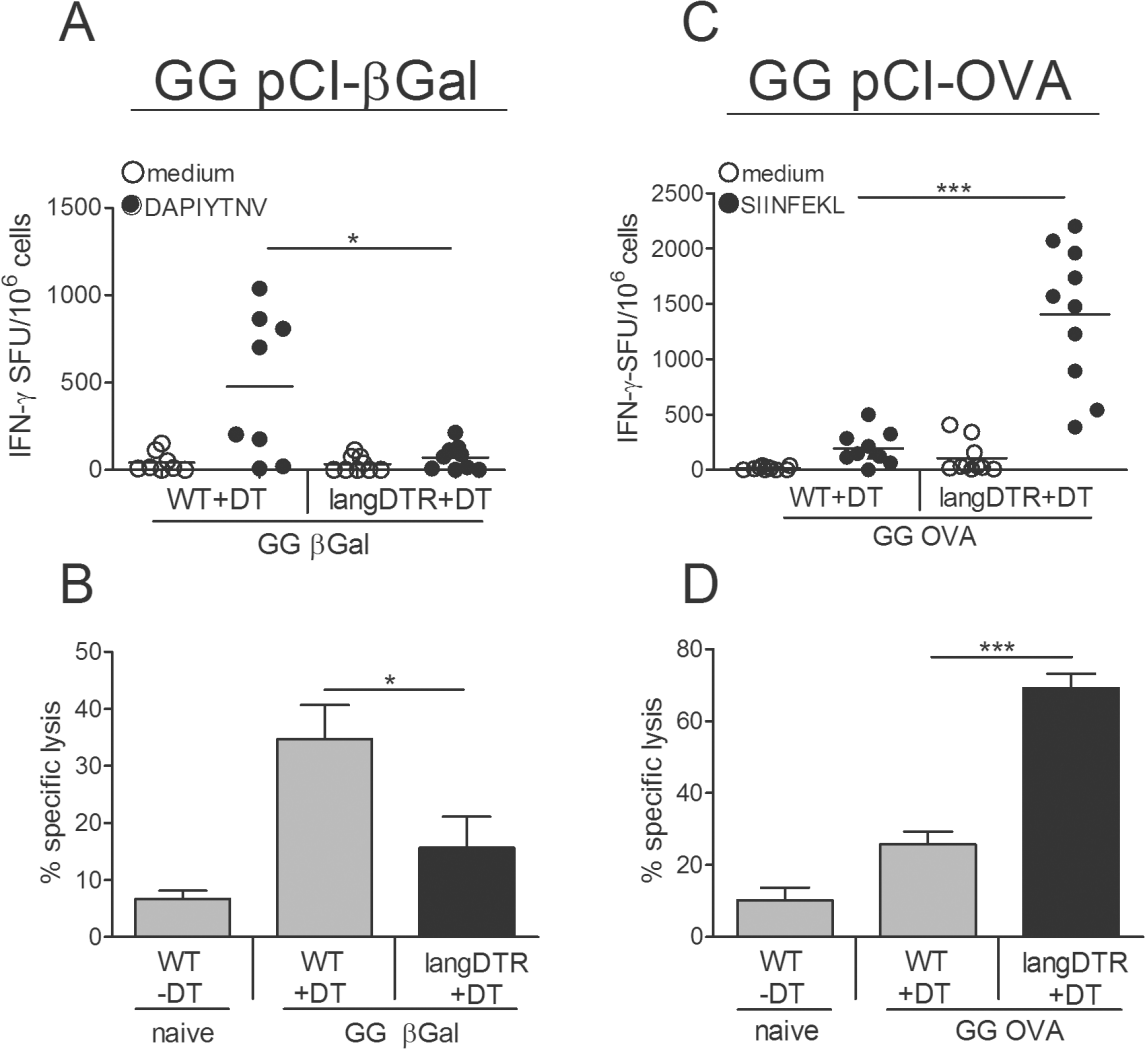
Depending on the antigen, langerin^+^ DC enhance or suppress CD8+ T cell responses. WT or langDTR mice were given DT 5x at 3d intervals, starting 1 d before immunization. Mice were GG-immunized twice at a 1wk interval with pCI-βGal or pCI-OVA. One week after the boost, splenocytes were restimulated in vitro with CTL peptides (A) DAPIYTNV for βGal or (C) SIINFEKL for OVA, and analyzed for IFNγ secretion by ELISPOT. Specific lysis of CTL peptide-pulsed target cells injected into (B) βGal- or (D) OVA-immunized mice 1 wk after the boost. WT mice without DT served as naïve controls. Means ± SD of cumulative data of two independent experiments with 4–5 mice, each.

In a previous study we found that the absence of langerin^+^ DC resulted in a strong reduction of CD8^+^ T cell responses to a βGal gene gun vaccine [18]. To examine whether other antigens would also require langerin^+^ DC for CTL activation we tested a DNA vaccine encoding chicken ovalbumin (pCI-OVA). Continuous ablation of all langerin^+^ DC was achieved in langDTR mice by repetitive diphtheria toxin (DT) injections at 3 day intervals [18]. Depleted and non-depleted mice were GG-immunized with pCI-βGal or pCI-OVA one day after DT treatment and boosted one week later. One week after the boost, systemic effector functions of CD8^+^ T cells were analyzed in the spleen of mice, immunized with the respective plasmids. As expected, with pCI-βGal, IFNγ-producing CD8^+^ T cells were hardly above background and cytotoxic activity was much weaker in DT-treated langDTR than in wild type (WT) mice (Fig 1A and 1B). Surprisingly, these effects were totally reversed in mice GG-immunized with pCI-OVA. In the absence of langerin^+^ DC the number of IFNγ-secreting CD8^+^ T cells and cytotoxic activity were strongly enhanced, rather than suppressed (Fig 1C and 1D). This is in apparent conflict with the strongly reduced proliferative response of adoptively transferred OT-1 T cells in langerin^+^ DC-depleted mice [18], which may originate from biological as well as technical differences in the experimental settings (S1 File). Thus, from above data we conclude that langerin^+^ DC were able to, either, promote or suppress CTL activation, and this modulating function seemed to be controlled by the nature of the antigen. Because CMV promoter-driven vaccines are active in all cell types, CD8^+^ T cells could have been activated, either, by directly transfected DC or via cross-presentation. However, the same results were obtained with keratinocyte-restricted DNA vaccines that strictly require cross presentation (S1 Fig). Thus, depending on the antigen, CD8^+^ T cells were apparently primed by different DC subtypes with cross-presenting capacity, i.e. langerin^+^ DC for βGal and langerin^neg^ DC for OVA.

### Depending on the antigen, langerin^+^ DC enhance or suppress antibody production

**Fig 2:**
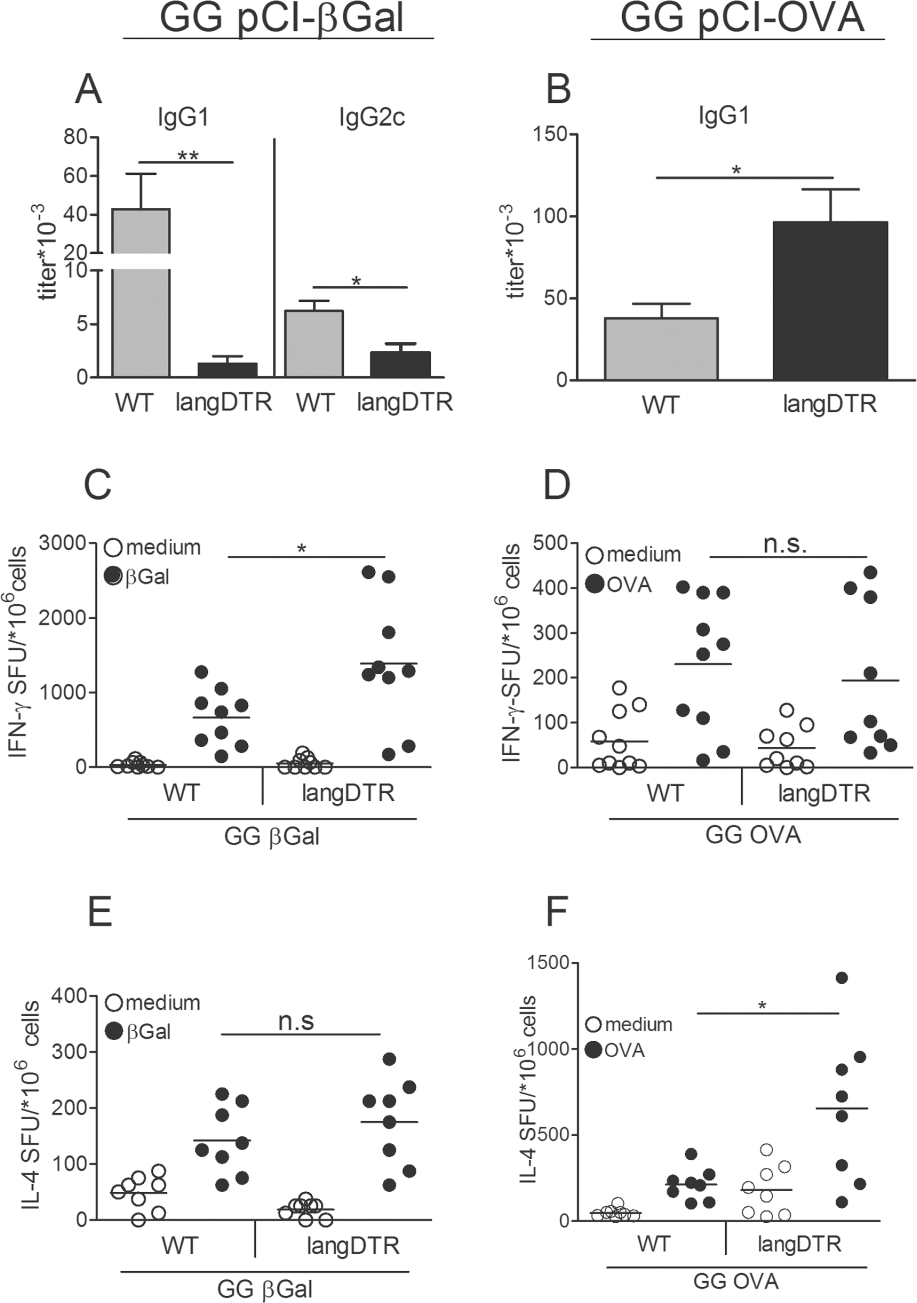
Depending on the antigen, langerin^+^ DC enhance or suppress antibody production. LangDTR or WT mice were treated 5x with DT at 3d intervals, starting 1 d before GG immunization. Mice received two GG immunizations with pCI-βGal or pCI-OVA at a 1-wk interval. Naïve WT mice without DT treatment served as controls. (A, B) serum IgG1 or IgG2c was analyzed by ELISA 1wk after the boost. IFNγ (C, D) and IL4 (E, F) ELISPOT assays of spleen cells of the same mice, restimulated with βGal or OVA protein, or medium. Means ± SD of cumulative data of two independent experiments with 4–5 mice, each.

We also wished to examine how the absence of langerin^+^ DC might affect antibody production. In mice, GG vaccines usually induce IgG1 as the predominant antibody isotype. In contrast to many other antigens, including OVA, βGal induces also other IgG subtypes [19, 28]. Testing serum samples of the mice described in Fig 1 revealed that ablation of langerin^+^ cells caused a dramatic reduction in IgG1 against βGal (Fig 2A, left panel) and, to a lesser extent, also a decline in IgG2c antibodies (Fig 2A, right panel). In striking contrast, OVA-specific IgG1 was strongly elevated in mice lacking langerin^+^ DC (Fig 2B). However, IgG2c antibodies against OVA were never detected (data not shown). The polarization of T helper (Th) cells was investigated by ELISPOT technique, thereby restimulating spleen cells with the respective protein antigens to enable the activation of CD4^+^ T cells. Ablation of langerin^+^ DC promoted IFNγ but not IL4-secreting T cells in βGal-immunized mice (Fig 2C and 2E). By contrast, in OVA-immunized mice IL4 but not IFNγ-secreting T cells were enhanced in the absence of langerin^+^ DC (Fig 2D and 2F). Thus, langerin^+^ DC either enhanced or suppressed IgG antibody production and modulated Th cell polarization, and the way they did was controlled by the respective antigen.

### Epidermal LC are dispensable for immunity against βGal but suppress CTL and modulate CD4^+^ T cell reactions against OVA

**Fig 3:**
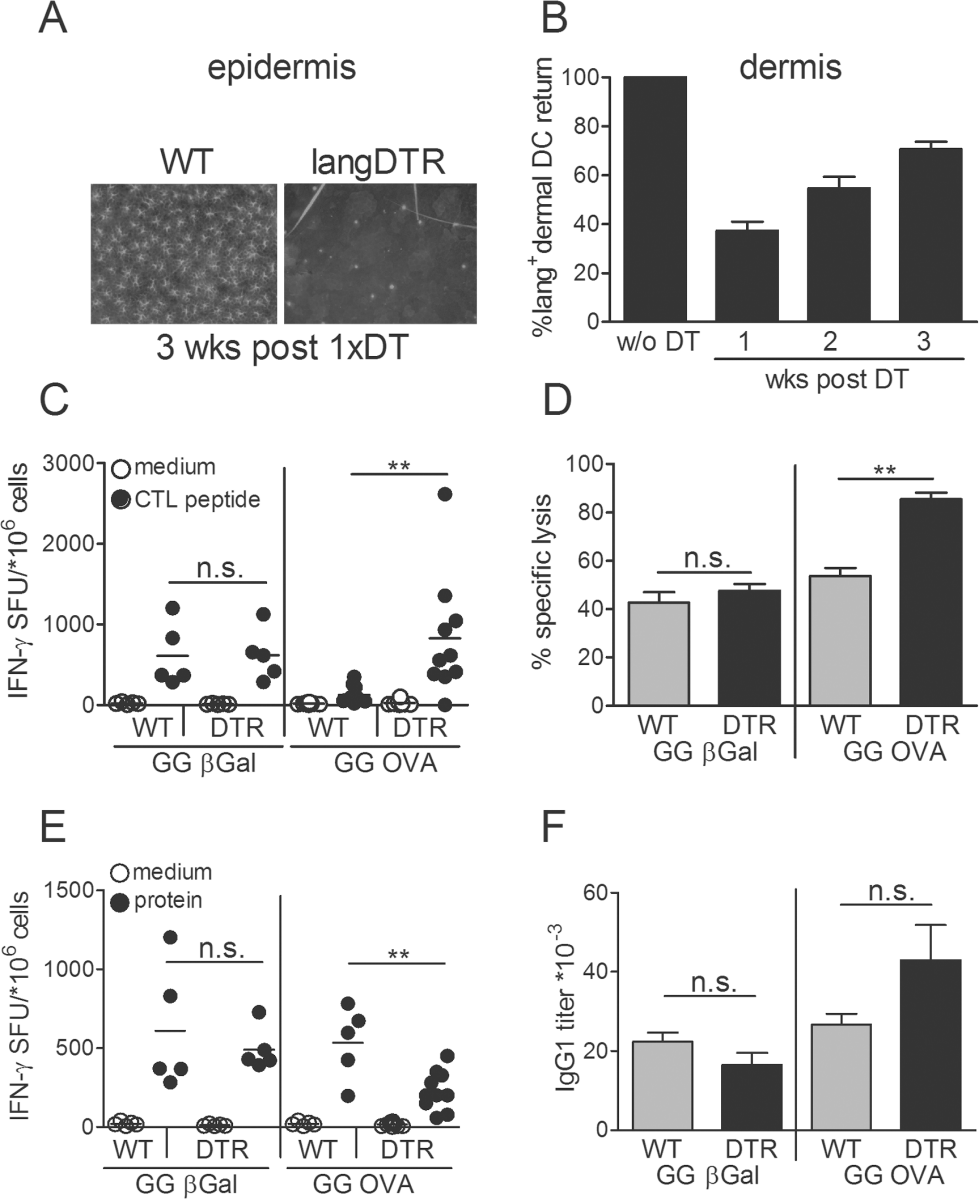
Epidermal LC are dispensable for βGal-specific immunity but down-regulate OVA-specific T cell functions. LangDTR or B6 WT mice were injected once with 1μg DT. Epidermal sheets were stained for langerin expression 3 wks later. (B) Repopulation of langerin^+^ DC in the dermis analyzed by FACS. LdDC from back skin were identified as CD11c^+^ EpCam^neg^ langerin^+^ cells. Groups of langDTR and B6 WT mice, treated once with 1μg DT were GG-immunized with either, pCI-OVA or pCI-βGal starting 1wk after DT treatment and boosted after 1wk. Splenocytes were in vitro restimulated with (C) CTL peptides DAPIYTNV for βGal- (left panel) or SIINFEKL for OVA-immunized mice (right panel) or with (E) βGal protein (left panel) or OVA protein (right panel) and analyzed for IFNγ secretion by ELISPOT technique. (D) Specific lysis of DAPIYTNV-pulsed (left panel) or SIINFEKL-pulsed (right panel) syngeneic target cells injected into GG-immunized langDTR and WT mice. (F) IgG1 antibody titers were analyzed by ELISA. Data represent means ± SD of groups of 4–5 mice and representative at least of two experiments.

In order to investigate specifically the contribution of epidermal LC on antigen-specific immune modulation, we took advantage of the differential repopulation kinetics of LdDC and LC. After DT-mediated ablation, LdDC reappear within a few days whereas the epidermis stays devoid of LC for several weeks [5]. To allow for repopulation of the skin with LdDC, GG immunization was started not immediately but, rather, 1week after a single DT injection, and mice were boosted again 1week later. This regimen provided a time window of 2 weeks where antigen presentation was accomplished without the involvement of epidermal LC but in the presence of LdDC that, at least partially, repopulated the skin within this time period (Fig 3A and 3B).For βGal, LC deficiency had no effect on IFNγ secretion and cytolytic activity of CD8^+^ T cells (Fig 3C and 3D left panel), and also not on IgG1 titers in serum or IFNγ production by spleen cells restimulated with protein antigen (Fig 3E and 3F left panel). However, in mice immunized against OVA, LC depletion increased IFNγ secretion in response to peptide restimulation (Fig 3C, right panel) and cytotoxic activity (Fig 3D right panel). Interestingly, contrary to the Th2 bias of the anti-OVA response seen after depletion of all langerin^+^ DC (elevated IL-4 secretion, Fig 2F), deficiency in only the LC compartment skewed the CD4^+^ T cells response towards Th1, as evidenced by higher IFNγ secretion (Fig 3E right panel) but comparable IL-4 (not shown) and IgG1 production (Fig 3F right panel). Together with the findings from the above sections we concluded that LdDC but not LC are required for anti-βGal responses. For anti-OVA responses, however, epidermal LC suppress CD8^+^ T cell functions. The differences seen in OVA-specific Th polarization after depletion of all langerin^+^ DC or only the LC compartment strongly suggest opposing functions of LC and LdDC. While LC (in concert with langerin^neg^ DC) promoted a Th2 cytokine profile, LdDC are required for the development of Th1 cells which in turn were able to counter regulate Th2 cell responses.

### βGal- and OVA gene gun vaccines react autonomously and do not influence each other

**Fig 4:**
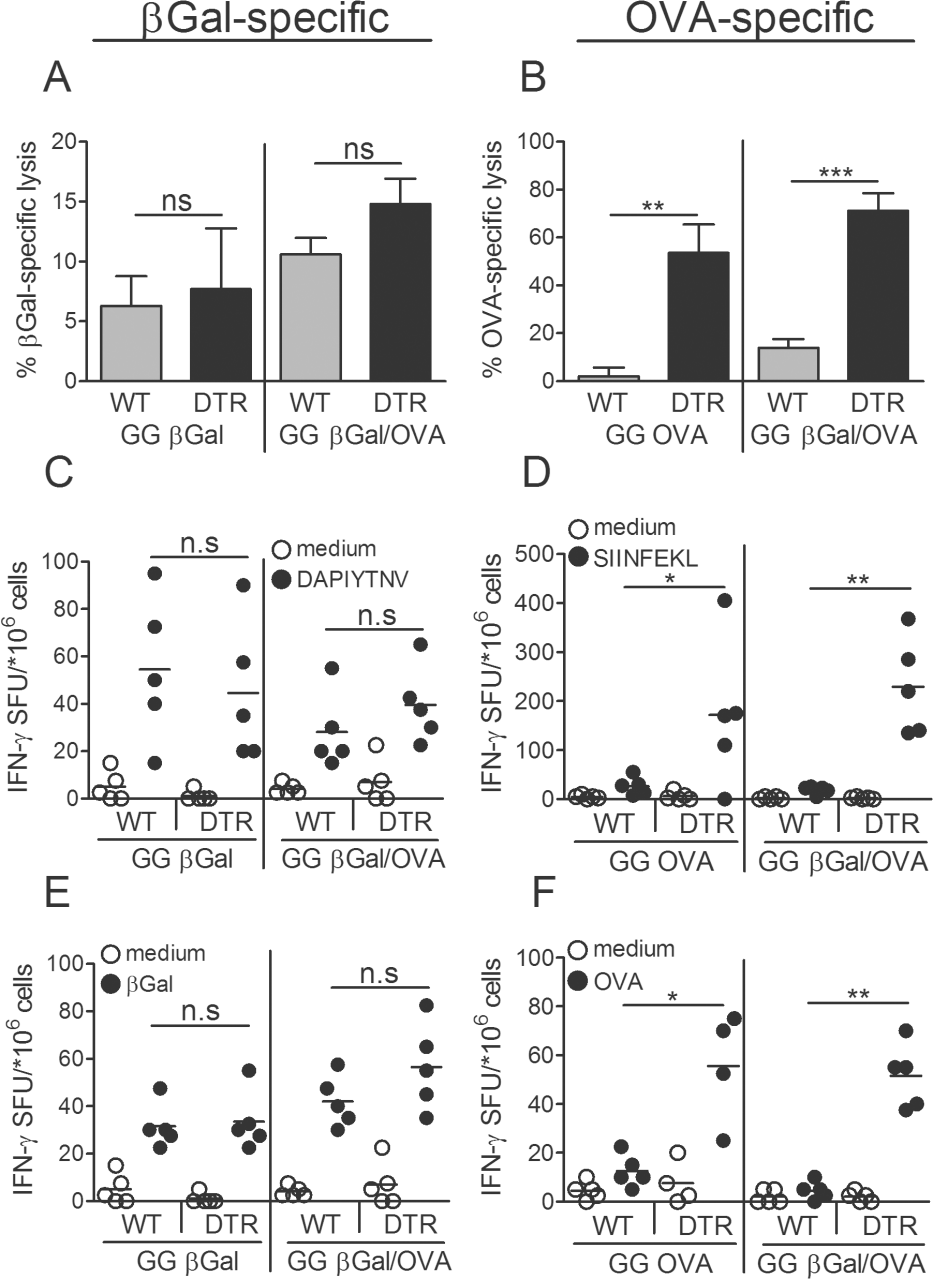
βGal- and OVA gene gun vaccines react autonomously and do not influence each other. LangDTR or B6 WT mice were injected once with 1μg DT and GG-immunized 1wk later with either pCI-βGal or pCI-OVA, or an equimolar mix of pCI-βGal/pCI-OVA plasmids co-precipitated onto the same gold particles. Mice were boosted 1 wk after the first immunization and analyzed 1wk later. Specific lysis of (A) DAPIYTNV-pulsed or (B) SIINFEKL-pulsed syngeneic target cells injected into GG-immunized langDTR and WT mice. Splenocytes were in vitro restimulated with (C) DAPIYTNV, (D), SIINFEKL, (E) βGal protein or (F) OVA protein and analyzed for IFNγ secretion by ELISPOT technique. Data represent means ± SD of groups of 5 mice and are representative of two experiments.

In view of the contrasting behavior of these two antigens we speculated if either of them might actively modulate the immune system. Modulation could be mediated, either, by features of the plasmid DNA or of the expressed protein antigen. The vaccine constructs pCI-βGal and pCI-OVA contain 44 or 22 immune-stimulatory sequence motifs with the canonical hexamer sequence RRCGYY [29], respectively, and might hence cause differential activation of DC. Alternatively, and not necessarily mutually exclusive, the translated antigens could influence the physiology of transfected cells differentially. To test these hypotheses we prepared a gene gun vaccine with a mixture of βGal and OVA-encoding plasmids precipitated onto the same gold particles. With this strategy, the same cells come in contact with both plasmids and may eventually be transfected by both plasmids. Hence, any modulating activity of one DNA vaccine (or its gene product, respectively) should influence the immune reaction against the other one. We used this mixed-plasmid vaccine to immunize wild type mice or mice selectively devoid of LC but not other langerin^+^ DC. With the mixed vaccine (Fig 4A–4F right panel), both antigens developed their characteristic immune reactions in the same way as when each of them was administered alone (Fig 4A–4F left panel). I.e., mice co-immunized with βGal/OVA responded to βGal with unaltered but to OVA with strongly enhanced T cell activity when LC were ablated. Thus, even when administered to the same cells, there was no cross talk between vaccines. Therefore, these findings suggest that the contrasting reactions induced by the two antigens were not due to immune-modulating activities of, either, the plasmid DNA or the antigen gene product.

### Antigen-specific immune modulation is not caused by differential cellular localization of the antigen

**Fig 5:**
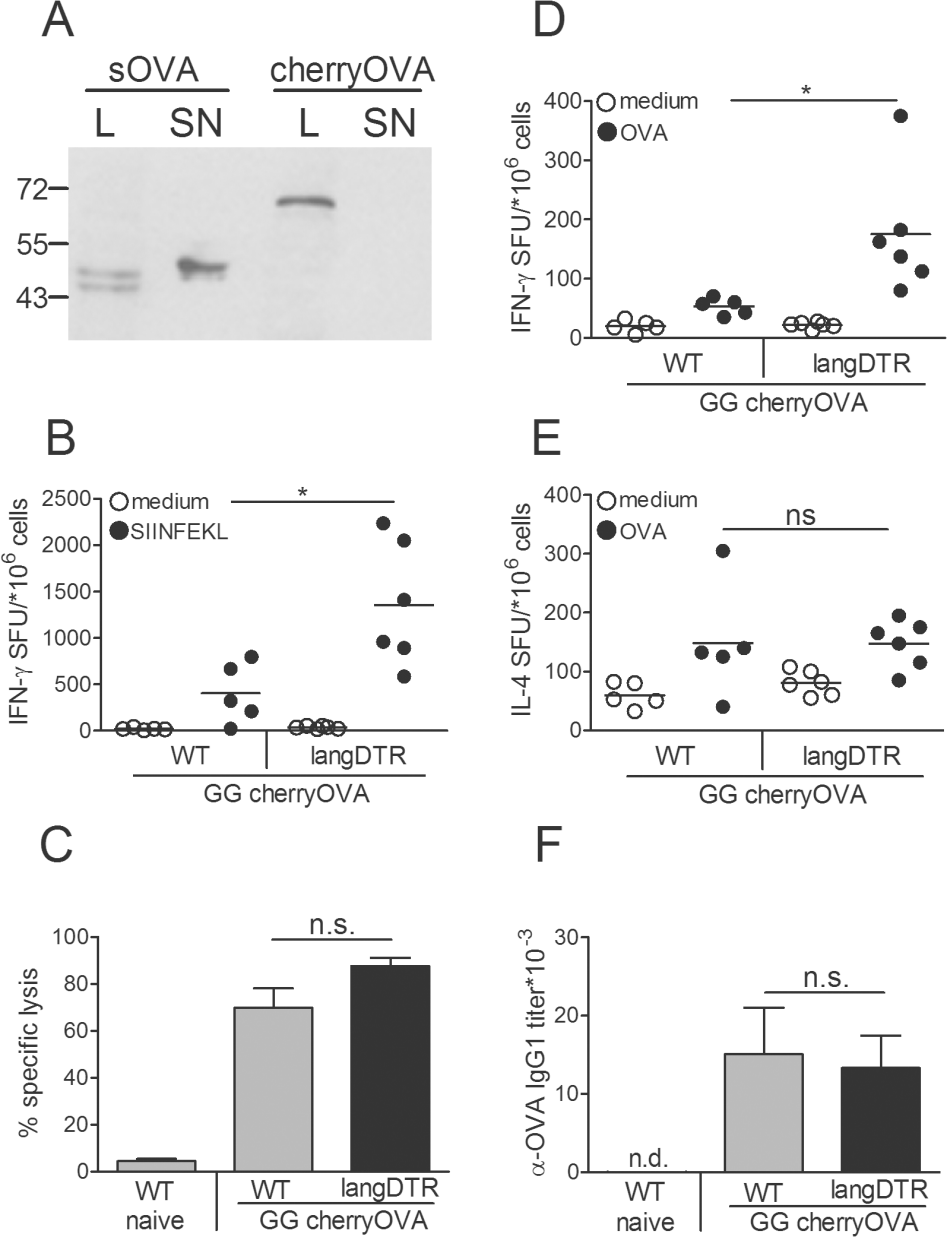
Antigen-specific immune modulation is not due to differential cellular localization of the vaccine gene product. (A) Western blots of cell lysates (L) and supernatants (SN) of BHK cells transfected with the respective gene vaccines pCI-OVA or pCI-cherryOVA. LC-depleted langDTR and DT-treated B6 WT mice were GG-immunized with pCI-cherryOVA twice at a 1 wk interval, starting 1wk after a single DT injection. One week after the boost splenocytes were restimulated in vitro with (B) SIINFEKL or (*D*, *E*) OVA protein and analyzed for (B, D) IFNγ and (E) IL4 secretion by ELISPOT technique. (C) Specific lysis of SIINFEKL-pulsed syngeneic target cells injected into langDTR and WT mice immunized with pCI-cherryOVA. (F) Serum IgG1 titer from pCI-cherryOVA-immunized mice was analyzed by ELISA. Data represent means ± SD of groups of 5 mice. Data shown are representative of two independent experiments.

One obvious difference between the two antigens is that βGal is retained in the cytosol whereas OVA is naturally secreted from transfected cells [30]. To examine whether the opposing effects of OVA and βGal GG-vaccines were related to this differential cellular localization of antigens we fused the coding sequence of OVA to the C-terminus of mCherry which prevented secretion of the gene product (Fig 5A). However, as observed before with secreted OVA, the cytosol-confined variant cherryOVA again yielded a more than three-fold increase in IFNγ-secreting CD8^+^ T cells of LC-depleted mice (Fig 5B). This effect was less clearly visible in the cytotoxic activity, primarily because of the strong CTL reaction that was already induced in the WT mice (Fig 5C). Moreover, as with secreted OVA, LC deficiency enhanced IFNγ production by CD4^+^ T cells also with the cytosol-retained fusion vaccine (Fig 5D), whereas IL4^+^ T cells and IgG1 titers did not change after LC ablation (Fig 5E and 5F). Collectively, these data indicate that the differential handling of antigens by distinct DC subsets is not a consequence of the fact that βGal is retained in the cytosol of transfected cells while OVA is secreted.

### Regulatory T cells are relevant for suppression of anti-OVA T cell responses

**Fig 6:**
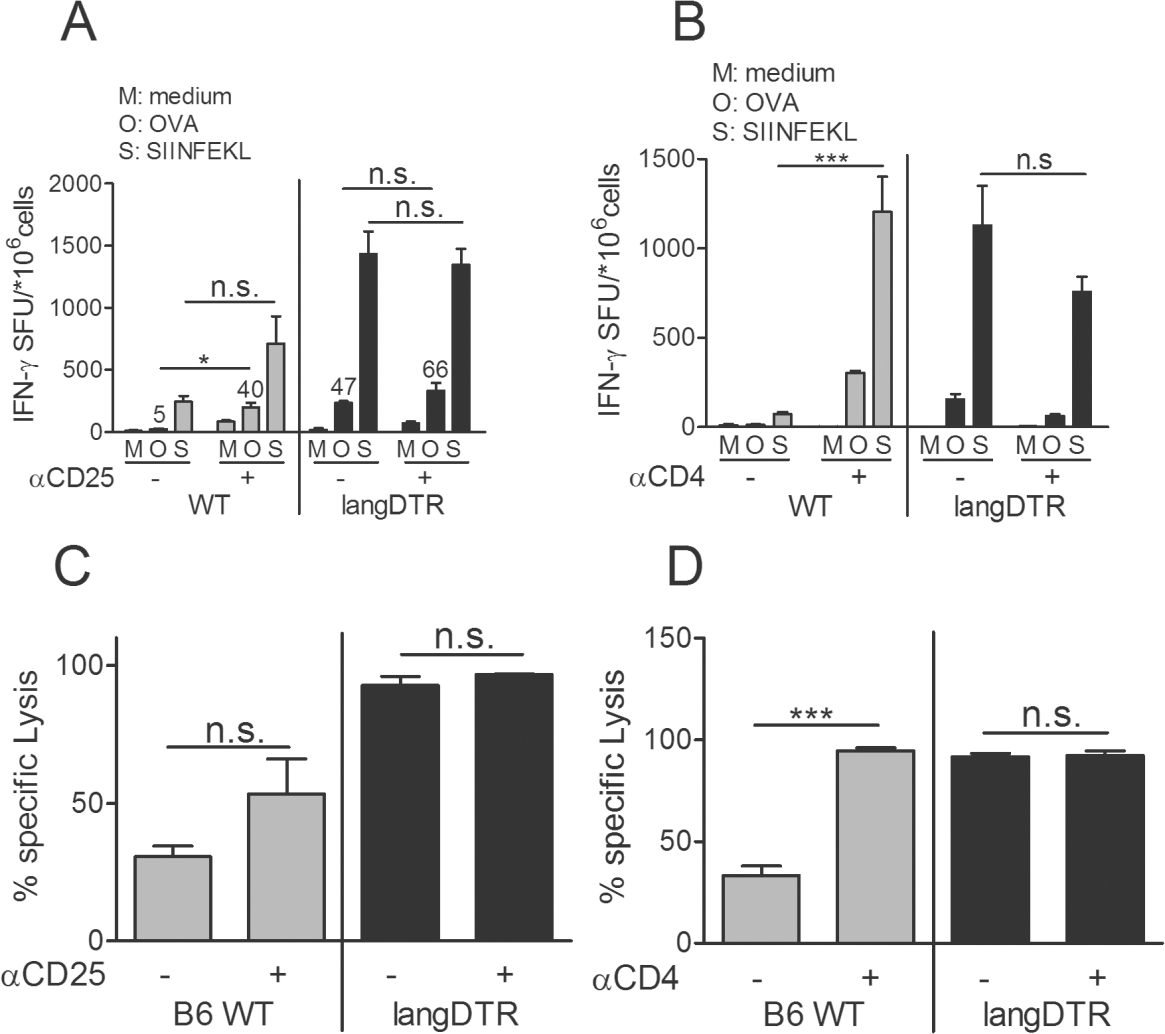
Regulatory T cells are relevant for suppression of OVA-specific T cell responses. LC-depleted langDTR and DT-treated B6 WT mice were injected three times with anti-CD25 Ab or anti-CD4 Ab (250μg /injection i.p.) at 5d intervals, starting 1day before GG immunization with pCI-OVA, or left without Ab injection. GG immunization was performed twice, with a 1wk interval. One week after the boost splenocytes of (A) CD25-depleted or (B) CD4-depleted mice, together with cell from non-depleted immunized controls, were restimulated in vitro with SIINFEKL (S) or OVA protein (O) or with medium (M) and analyzed for IFNγ secretion by ELISPOT technique. Specific lysis of SIINFEKL-pulsed syngeneic target cells injected into (C) CD25-depleted or non-depleted langDTR and WT mice or (D) CD4-depleted or non-depleted langDTR and WT mice. Data represent means ± SD of groups of 5–6 mice.

The elevated responses in LC-deficient mice that were observed with both, the secreted and cytosol-confined OVA variants suggested that epidermal LC might be involved in the suppression of immune responses. LC have been implicated in the suppression of immune reactions in other models before, with a potential role in the activation of regulatory T (T_reg_) cells [14, 16]. Therefore, we explored whether the exaggerated T cell responses to OVA in LC-deficient mice might be associated with impaired T_reg_ activation. If so, T_reg_ depletion in WT mice should elevate T cell responses to levels of LC-depleted mice but not further amplify the yet strong responses in the latter. When we treated WT mice with anti-CD25 Ab during GG vaccination (S2 Fig) the frequency of T cells that secreted IFNγ upon restimulation with OVA protein increased 8-fold (Fig 6A, left panel), close to that in LC-depleted mice. IFNγ secretion by CD8^+^ T cells and cytotoxic activity were also enhanced in CD25-depleted WT mice (Fig 6A and 6C left panels). However, they did not fully reach the level observed in LC-deficient mice (Fig 6A and 6C right panels) suggesting, either, collateral depletion of CD25^+^ Th effector cells or the existence of additional suppressors, e.g. CD25^neg/low^ T_reg_ cells [31, 32]. To investigate this, we repeated the above experiments with anti-CD4 Ab treatment during vaccination (S2 Fig). We hypothesized that, if Th cells were important, this treatment should result in a collapse of CTL. Alternatively, if CD25^neg/low^ T_reg_ cells were important CTL would increase beyond the levels observed with anti-CD25 treatment. After depletion of CD4^+^ T cells, CTL activity in WT mice reached a maximum and was now identical to that achieved with LC depletion (Fig 6B and 6D left panels). Importantly, in LC-deficient mice neither Ab could further increase the response (Fig 6A–6D, right panels). Thus, OVA-specific CTL activation was solely impaired when both cell types, LC and CD4^+^ T cells, were intact during GG vaccination. These data suggest that LC can induce both, CD25^+^ as well as CD4^+^CD25^neg/low^ T cells with the capacity to suppress CTL activation.

**Fig 7:**
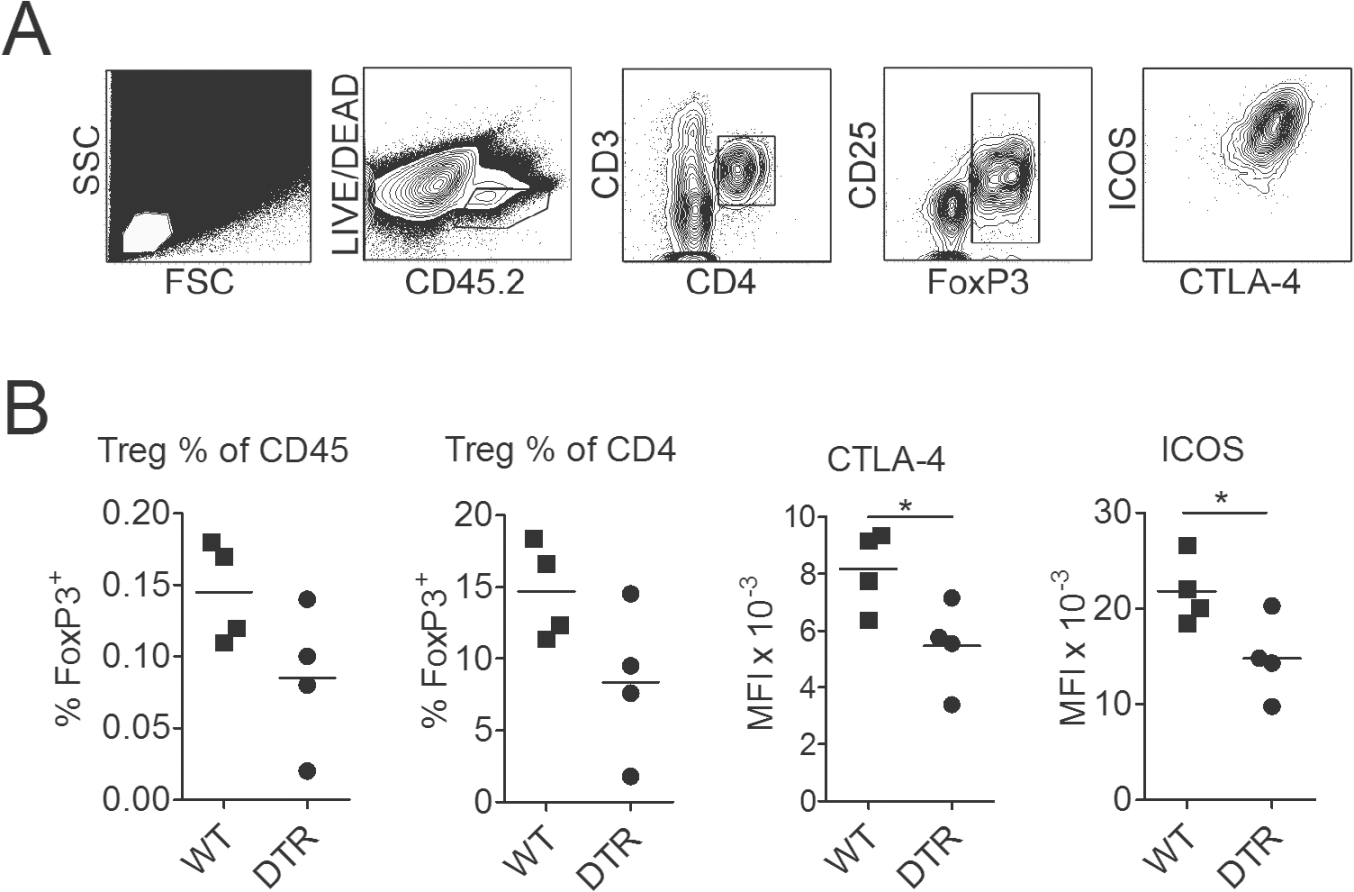
Activation and frequency of regulatory T cells are decreased in LC-depleted mice. Trunk skin lymphocytes in langDTR or WT mice (n = 4, each) were analyzed by flow cytometry three weeks after a single injection of 1μg DT. (A) Gating strategy: the lymphocyte gate in the leftmost panel was set according to the FSC/SSC properties found for LN cells (not shown). ICOS and CTLA-4 expression of CD4^+^FoxP3^+^ cells was read as mean fluorescence intensity (MFI). MFI was 285 for CTLA-4 and 165 for ICOS, respectively, in CD45^neg^ control non-lymphocytes. (B) Frequency of CD4^+^FoxP3^+^ cells in trunk skin of DT-treated langDTR (DTR) or WT (Bl/6) mice, expressed as % of CD45.2^+^ or CD4^+^ cells, respectively (left panels), and MFI of T_reg_ activation markers CTLA-4 and ICOS of the same cells (right panels). Statistical analysis was performed by two-tailed Student’s t-test.

To further investigate the effect of LC-deficiency on T_regs_ we analyzed trunk skin of langDTR or WT control mice. Three weeks after DT injection, the frequency of CD4^+^FoxP3^+^ T cells was decreased in LC-depleted mice, both, within the CD45^+^ leukocyte population as well as among CD4^+^ T cells. Moreover, T_regs_ in LC-depleted mice were significantly lower for CTLA-4 and ICOS surface expression as compared to WT controls (Fig 7). Thus, the presence of LC seemed to promote, both, the frequency as well as the activation status of T_regs_ in the skin.

## Discussion

The various DC subsets in the skin are functionally diverse and influence downstream immune effector functions in different ways. Much of this diversity originates from differential sensitivity to, and subset-specific orchestration of endogenous or pathogen-associated danger signals. In contrast, protein antigens and their role in shaping immune reactions has been largely left unnoticed. Here, we show that different skin-borne protein antigens may be handled differentially by distinct DC subsets and, in this way, contribute to the modulation of immune reactions by virtue of their intrinsic molecular features.

To study skin DC it is imperative to deliver the antigen in a way that restricts antigen localization to the skin. Therefore, we employed gene gun immunization with DNA vaccines. Importantly, with this method the administered substance is always plasmid DNA, irrespective of the antigen, thus, allowing for the investigation of different protein antigens under otherwise identical conditions. We compared the immune response to βGal with that to OVA and investigated their requirement for various DC subsets. In langerin^+^ DC-deficient mice CTL and IgG1 against βGal were impaired, whereas these functions were strongly enhanced in OVA-immunized animals. These findings impose three major implications:

First, the strong CTL response in the absence of langerin^+^ DC, which was also observed with a keratinocyte-restricted DNA vaccine, pK14-OVA, indicated that a langerin^neg^ DC subset was capable of cross-presenting antigen to CD8^+^ T cells. This was interesting because, among skin DC, this competency had initially been attributed specifically to CD103^+^ LdDC [8, 33]. Meanwhile, however, more recent studies had also demonstrated antigen cross presentation by langerin^neg^ skin DC in various experimental settings, including C. albicans infection [15], adenovirus-based vaccines [34], or topical administration of antigen onto the skin [35]. Thus, our data add further evidence for antigen cross presentation by langerin^neg^ skin DC.

Second, epidermal LC (or their progeny in LN) suppressed GG-induced anti-OVA reactions. This was deduced from the observation that CTL and antibody titers were elevated under conditions where the slower repopulating LC were still absent while langerin^+^ DC with higher turnover rates had repopulated tissues; data that were also confirmed in an alternative mouse model [9] that allows for the selective ablation of LC, i.e. with all other langerin^+^ DC not being affected by DT (S3 Fig). LC-dependent immunosuppression and/or induction of tolerance has also been shown in experimental settings of leishmania infection, CHS or skin transplantation [14, 16, 36, 37]. Depending on the model, the involvement of T_regs_ has been demonstrated or suggested but other mechanisms, such as the direct killing or inactivation of CTL by engagement of LC-derived FasL, or IL10 secretion by LC have also been discussed [37]. In our model, LC-deficiency caused a decrease in the frequency of T_reg_ cells and their activation status. It seems conceivable that this contributes to the elevated CTL response in LC-depleted mice against OVA GG vaccines. In line with this, CTL responses in WT mice were enhanced by depletion of CD25^+^ T cells. However, the full level of CTL activation that was seen in LC-deficient mice was only achieved after depletion of the entire CD4^+^ T cell compartment. Importantly, neither of these treatments had any additive effect to CTL in LC-ablated mice, suggesting that LC- and T cell-mediated suppression are not independent mechanisms but part of the same pathway. From this, we conclude that LC control the magnitude of GG-induced CTL reactions against OVA by a mechanism that involves both, CD25^+^ and CD4^+^CD25^-^ T cells.

Third, intrinsic molecular features of skin-borne protein antigens seem to determine by which DC subsets they are presented, and also which effector functions these DC elicit. Our experiments revealed fundamental differences between the immune reactions induced by βGal or OVA GG vaccines with varying DC compositions. In summary, CTL responses against βGal strongly relied on LdDC but not LC. In contrast, CTL responses against OVA required langerin^neg^ DC but were suppressed by LC. Intriguingly, Th cell polarization was also regulated differentially, as the presence of LdDC suppressed IFNγ in βGal-immunized mice but IL-4 in OVA-immunized animals.

The differences between these antigens did not simply originate from the fact that OVA, in contrast to βGal, is secreted from transfected cells because anti-OVA reactions remained unaltered when secretion was prevented by fusion to mCherry. This also excluded differences in the molecular weight of antigens as a reason for their differential behavior. In contrast to secretory OVA, the fusion product exceeded the threshold of 70 kDa for size-controlled transmigration of lymph-borne molecules into LN cortical areas and conduits [38, 39]. Even when fused to βGal, OVA elicited stronger CTL responses in the absence of LC (S4 Fig). It is also unlikely that differences in the expression level accounted for the opposing effects, since transfection of keratinocytes in vitro with βGal or Cherry-OVA yielded similar amounts of protein (S5 Fig). Moreover, CTL reactions were still enhanced in LC-deficient mice when GG-immunized with suboptimal doses of pCI-OVA (S5 Fig), and also with a βGal-OVA fusion that enforces exactly identical molarities (S4 Fig). Further, we exclude differences in immune-stimulatory CpG motifs as a mechanism because co-administration of both plasmids on the same particles did not alter their differential behavior even though, in this setting, affected cells were exposed to the same DNA composition. From these findings, we conclude that hitherto undefined features that are intrinsic to the protein antigen influence the magnitude and type of effector mechanisms by differential engagement of distinct DC subsets.

The mechanisms by which antigens might be differentially selected for presentation by distinct DC subsets are still elusive. It has been proposed that DC can take up antigens not only by relatively unspecific processes such as macropinocytosis, but also receptor-mediated incorporation [40]. This would provide a basis for a molecular explanation, but remains speculation until receptors for specific antigens are identified. Generally, it is questionable whether the observed differences between antigens originate from differential access to antigen by distinct DC subsets at all. At least one observation argues for alternative or additional mechanisms. With the βGal GG vaccine langerin^neg^ DC activated CD4^+^ T cells, indicating that these DC must have had access to the antigen. Nevertheless, they were unable to induce βGal-specific CTL while, with the OVA GG vaccine, the same DC induced both, CD8^+^ and CD4^+^ T cells. However, langerin^neg^ DC do not comprise a homogeneous population but split up into further subsets [8] that could still have differential access to skin-borne antigens. Alternatively, βGal and OVA could be differentially processed within the same cell, perhaps analogously to the segregation of microbial and apoptotic material into distinct phagosomes [41]. Some antigens seem to actively modulate the physiology of immune cells, such as the house dust mite allergen Der p 2 which is a homolog of the LPS-binding Tlr4 co-receptor MD2 [42]. For βGal and OVA this seems unlikely because co-expression of both antigens in the same cell, did not result in any interference between immune reactions.

In conclusion, our data revealed a differential selectivity of skin DC for protein antigens and demonstrate that this contributes to the modulation of immune effector functions. Screening a larger panel of antigens, together with the investigation of other methods for antigen administration, could clarify whether antigen selectivity is a general feature of skin DC. This would have important implications for the understanding of skin immunity, diseases and vaccine development.

## Supporting Information

S1 File Gene gun-induced proliferation of adoptively transferred OT-1 cells is delayed in mice lacking langerin^+^ DC. (DOCX)

S1 Fig Langerin^+^ DC enhance or suppress CD8^+^ T cell responses against keratinocyte-derived antigens. WT or langDTR mice were given DT 5x at 3d intervals, starting 1 d before immunization. Mice were GG-immunized twice at a 1wk interval with K14-βGal or K14-OVA. One week after the boost, splenocytes were restimulated in vitro with CTL peptides (A) DAPIYTNV for βGal or (C) SIINFEKL for OVA and analyzed for IFNγsecretion by ELISPOT. Specific lysis of CTL peptide-pulsed target cells injected into (B) βGal- or (D) OVA-immunized mice 1 wk after the boost. WT mice without DT served as naïve controls. Data are means ± SD of 4–5 mice.
(TIF)

S2 Fig Depletion of CD25^+^ or CD4^+^ cells by i.p. injection of mAbs. LC-depleted LangDTR and DT-treated B6 WT mice shown in Fig 6 were injected three times with (A) anti-CD25 Ab or with (B), anti-CD4 Ab (250μg /injection i.p.) at 5d intervals, or left untreated. Success of cell depletion was analyzed by flow cytometry of spleen cells on the day of terminal analysis.
(TIF)

S3 Fig Selective Langerhans cell depletion in hu-langDTR mice enhances anti-OVA but not anti-βGal CTL responses. Groups of hu-langDTR mice were injected once with 1μg DT or were left untreated. One wk later, mice were GG-immunized with, either pCI-OVA or pCI-βGal twice at a one wk interval. One wk after the boost, splenocytes were restimulated in vitro with CTL peptides (A) SIINFEKL for OVA or DAPIYTNV for βGal and analyzed for IFNγ secretion by ELISPOT. (B) Specific lysis of CTL peptide-pulsed target cells injected into pCI-OVA or pCI-βGal-immunized mice. Data are means ± SD of 4–5 mice.
(TIF)

S4 Fig Immune modulation is not due to the size of the antigen. LangDTR or B6 WT mice were injected once with 1μg DT and GG-immunized 1wk later with a pCI-βGalOVA fusion plasmid that was generated by inserting the open reading frame of OVA right behind the last coding triplet of βGal. Mice were boosted 1 wk after the first immunization and analyzed 1wk later. (A) Splenocytes were in vitro restimulated with SIINFEKL or medium and analyzed for IFNγ secretion by ELISPOT. (B) Specific lysis of SIINFEKL-pulsed syngeneic target cells injected into GG-immunized langDTR and WT mice. Data represent means ± SD of groups of 5 mice and are representative of two experiments.
(TIF)

S5 Fig Differential immune responses were not caused by differences in antigen expression. (**A**) Western Blot of Pam212 keratinocytes transfected with the indicated doses of pCI-βGal or pCI-cherryOVA and cultured for two days. Blots were incubated with polyclonal anti-βGal or anti-OVA antisera, respectively, followed by peroxidase-labeled secondary antibody and subsequent luminogenic development. Luminescence was recorded on ChemiDoc MP imaging system (BioRad). The uppermost band in the right panel corresponds to the full length fusion product with an expected MW of 74 kDa. The second-largest band could result from premature translation stop or degradation of the gene product; lower bands are probably unspecific signals that are equally intense in non-transfected cells. Lower Panel: SDS-gel stained with Coomassie Blue after blotting is shown as a loading control. (**B**) LangDTR or B6 WT mice were injected once with 1μg DT and GG-immunized 1wk later with different doses of pCI-OVA (100, 300,1000ng plasmid/GG shot). Mice were boosted with the same doses after 1 wk and analyzed 1wk later. Specific lysis of SIINFEKL-pulsed syngeneic target cells injected into GG-immunized langDTR and WT mice. Data represent means ± SD of groups of 5 mice and are representative of two experiments.
(TIF)
