# Supplementary material for: Immune Reactions against Gene Gun Vaccines Are Differentially Modulated by Distinct Dendritic Cell Subsets in the Skin
Source: PLoS One. 2015 Jun 1;10(6):e0128722. doi: 10.1371/journal.pone.0128722 (PMC4452175; doi:10.1371/journal.pone.0128722)
Supplement: S1 File — (DOCX) [file pone.0128722.s001.docx]

**S1 File: Reduced Response OT-1 T cells in langerin+ DC-ablated mice.**



S1 Figure: Gene gun-induced proliferation of adoptively transferred OT-1 cells is delayed in mice lacking langerin^+^ DC. Groups of LangDTR and WT mice were treated once with 1 µg DT and, on the same day, received a 1:1 mixture of CFSE-labeled OT-I and WT splenocytes. Next day, mice were gene gun immunized with pCI-OVA and, at indicated time points, proliferation of viable OT-I cells was analyzed in SDLN by flow cytometry. (A) Representative dot plots of OT-1 expansion in SDLN of WT (upper panel) and langDTR mice (lower panel). (B) Time course of OT-I cell expansion calculated by normalizing OT-1 cell numbers to that of injected WT cells. Data are means ± SD of groups of five mice.

In langerin^+^ DC-ablated mice, we consistently observe severely impaired proliferation of adoptively transferred OT-1 T cells but enhanced OVA-specific responses of endogenous CD8+ T cells. While this opposing behavior is intriguing it is clear that examination of endogenous CD8+ T cell responses is the less artificial setting and reflects more authentically the natural course of an immune response. Nevertheless, it may be instructive to contemplate potential reasons:

1. With adoptively transferred OT-1 cells, only the clonal expansion was evaluated whereas the measurement of endogenous antigen-specific CD8+ T cells was based on functional parameters such as IFNγ production in ELISPOT assays, or cytolytic activity. Thus, differences in the measured parameters complicate direct comparison of results.
2. OT-1 cells are extremely sensitive and respond to very low concentrations of antigen. Also, they require little additional signals for activation; in vitro, formaldehyde-fixed DC pulsed with CTL peptide are sufficient to induce proliferation (Choi J. et al. (2009) J.Exp.Med. 206:497). Although somewhat speculative at this point, it is conceivable that the reduced OT-1 response primarily reflects the reduction in DCs that present cognate CTL peptide in LC-ablated mice whereas naïve endogenous CD8+ T cells, because they rely on additional parameters, benefit from the ablation of suppressive LC.
3. Compared to the very low number of endogenous antigen-specific T cells in a naïve mouse, the number of adoptively transferred OT-1 T cells (typically in the range of 10*6) is high beyond every physiological value. Thus, while in the endogenous setting, the immune response is launched from authentic starting conditions OT-1 transfers reflect conditions as met, at best, at the peak of a very strong CTL response.
