# Supplementary figures and images for: Immune Reactions against Gene Gun Vaccines Are Differentially Modulated by Distinct Dendritic Cell Subsets in the Skin
Source: PLoS One. 2015 Jun 1;10(6):e0128722. doi: 10.1371/journal.pone.0128722 (PMC4452175; doi:10.1371/journal.pone.0128722)

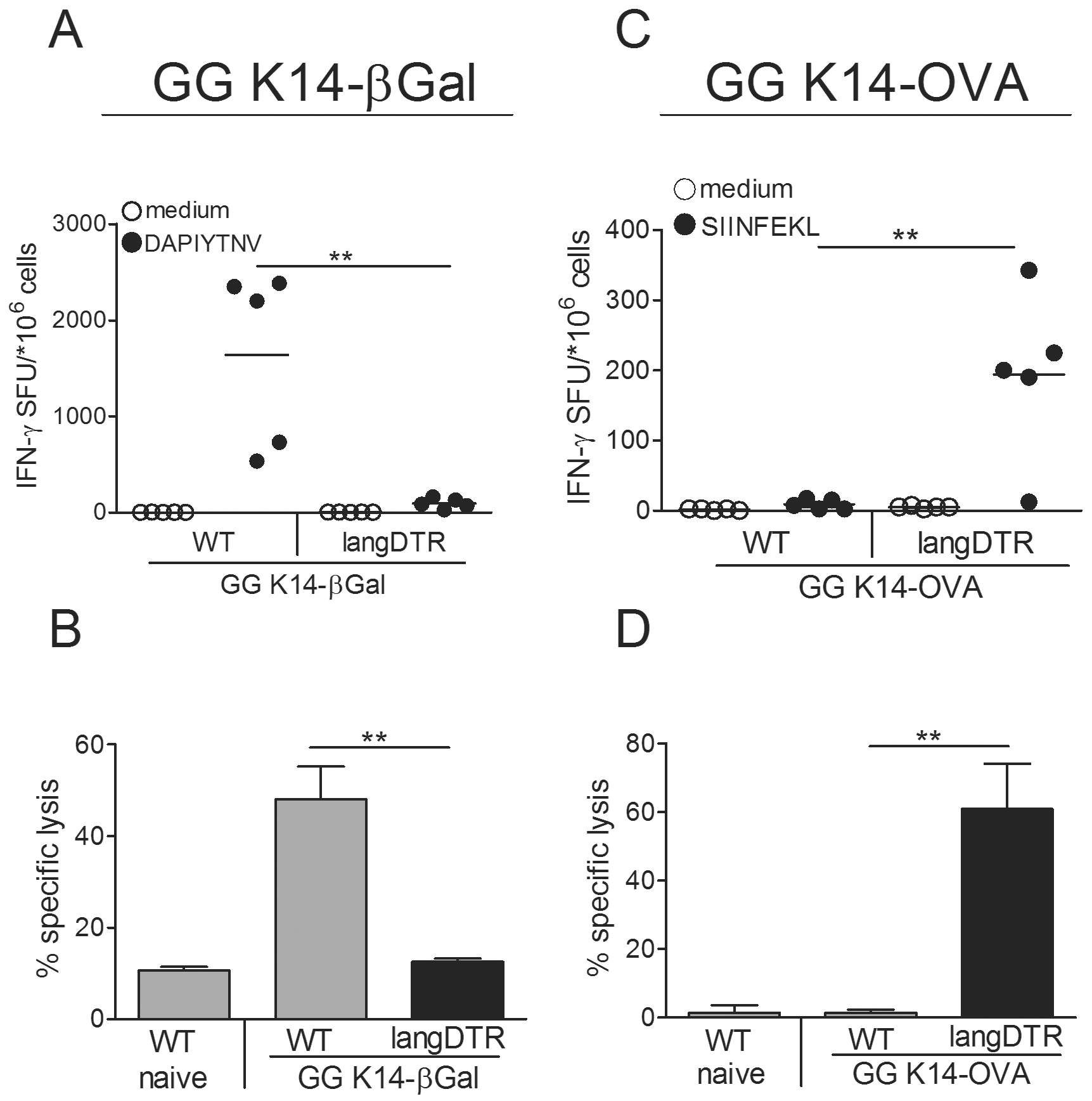

Supplement: S1 Fig — WT or langDTR mice were given DT 5x at 3d intervals, starting 1 d before immunization. Mice were GG-immunized twice at a 1wk interval with K14-βGal or K14-OVA. One week after the boost, splenocytes were restimulated in vitro with CTL peptides (A) DAPIYTNV for βGal or (C) SIINFEKL for OVA and analyzed for IFNγsecretion by ELISPOT. Specific lysis of CTL peptide-pulsed target cells injected into (B) βGal- or (D) OVA-immunized mice 1 wk after the boost. WT mice without DT served as naïve controls. Data are means ± SD of 4–5 mice. (TIF) [file pone.0128722.s002.tif]

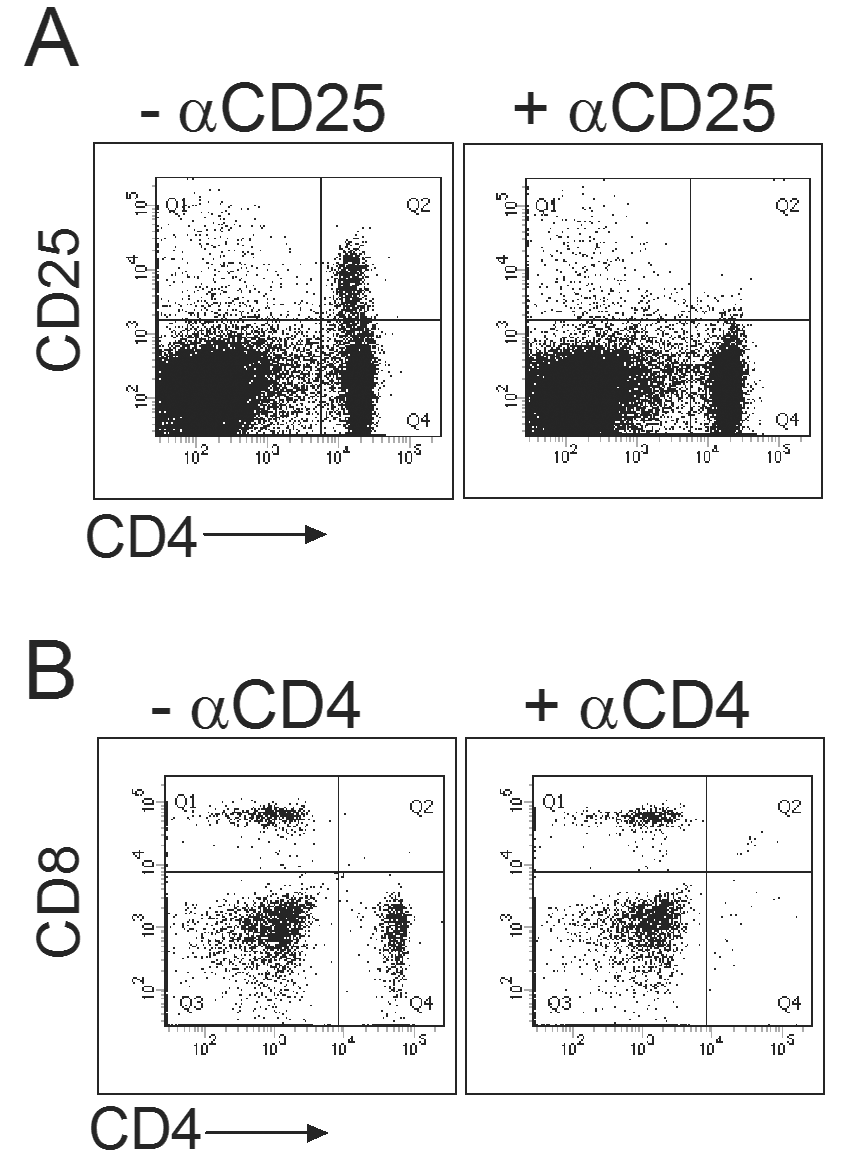

Supplement: S2 Fig — LC-depleted LangDTR and DT-treated B6 WT mice shown in Fig 6 were injected three times with (A) anti-CD25 Ab or with (B), anti-CD4 Ab (250μg /injection i.p.) at 5d intervals, or left untreated. Success of cell depletion was analyzed by flow cytometry of spleen cells on the day of terminal analysis. (TIF) [file pone.0128722.s003.tif]

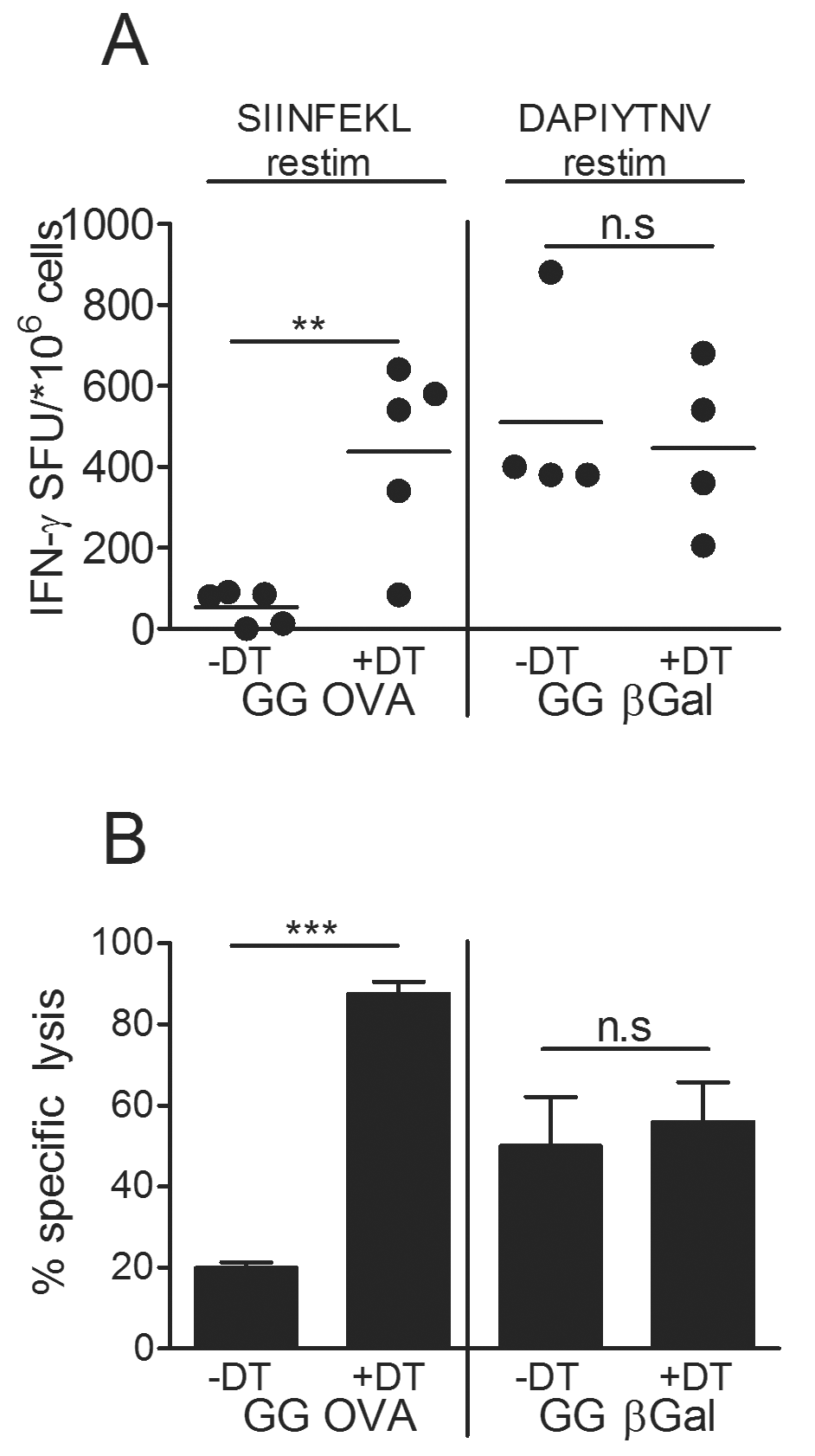

Supplement: S3 Fig — Groups of hu-langDTR mice were injected once with 1μg DT or were left untreated. One wk later, mice were GG-immunized with, either pCI-OVA or pCI-βGal twice at a one wk interval. One wk after the boost, splenocytes were restimulated in vitro with CTL peptides (A) SIINFEKL for OVA or DAPIYTNV for βGal and analyzed for IFNγ secretion by ELISPOT. (B) Specific lysis of CTL peptide-pulsed target cells injected into pCI-OVA or pCI-βGal-immunized mice. Data are means ± SD of 4–5 mice. (TIF) [file pone.0128722.s004.tif]

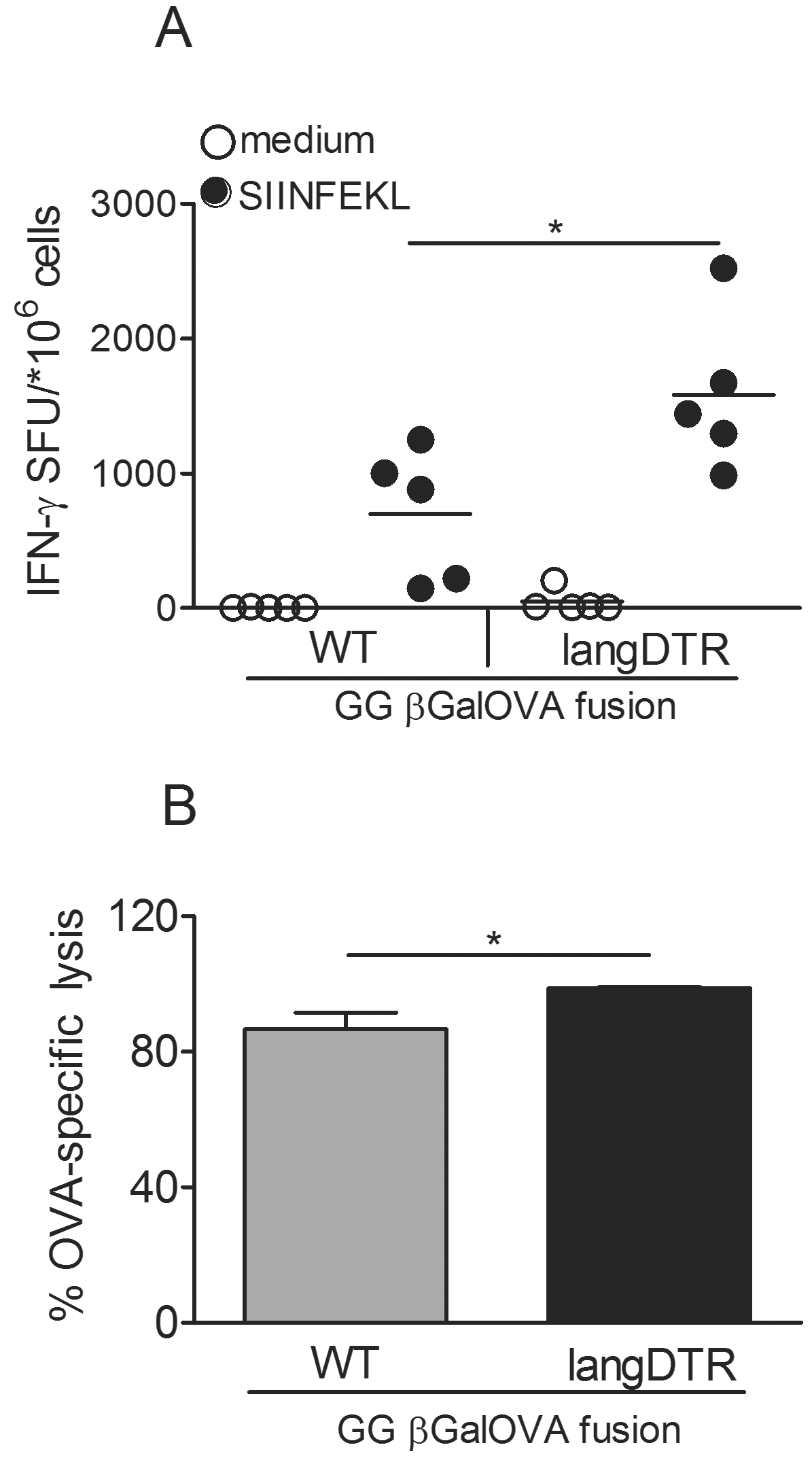

Supplement: S4 Fig — LangDTR or B6 WT mice were injected once with 1μg DT and GG-immunized 1wk later with a pCI-βGalOVA fusion plasmid that was generated by inserting the open reading frame of OVA right behind the last coding triplet of βGal. Mice were boosted 1 wk after the first immunization and analyzed 1wk later. (A) Splenocytes were in vitro restimulated with SIINFEKL or medium and analyzed for IFNγ secretion by ELISPOT. (B) Specific lysis of SIINFEKL-pulsed syngeneic target cells injected into GG-immunized langDTR and WT mice. Data represent means ± SD of groups of 5 mice and are representative of two experiments. (TIF) [file pone.0128722.s005.tif]

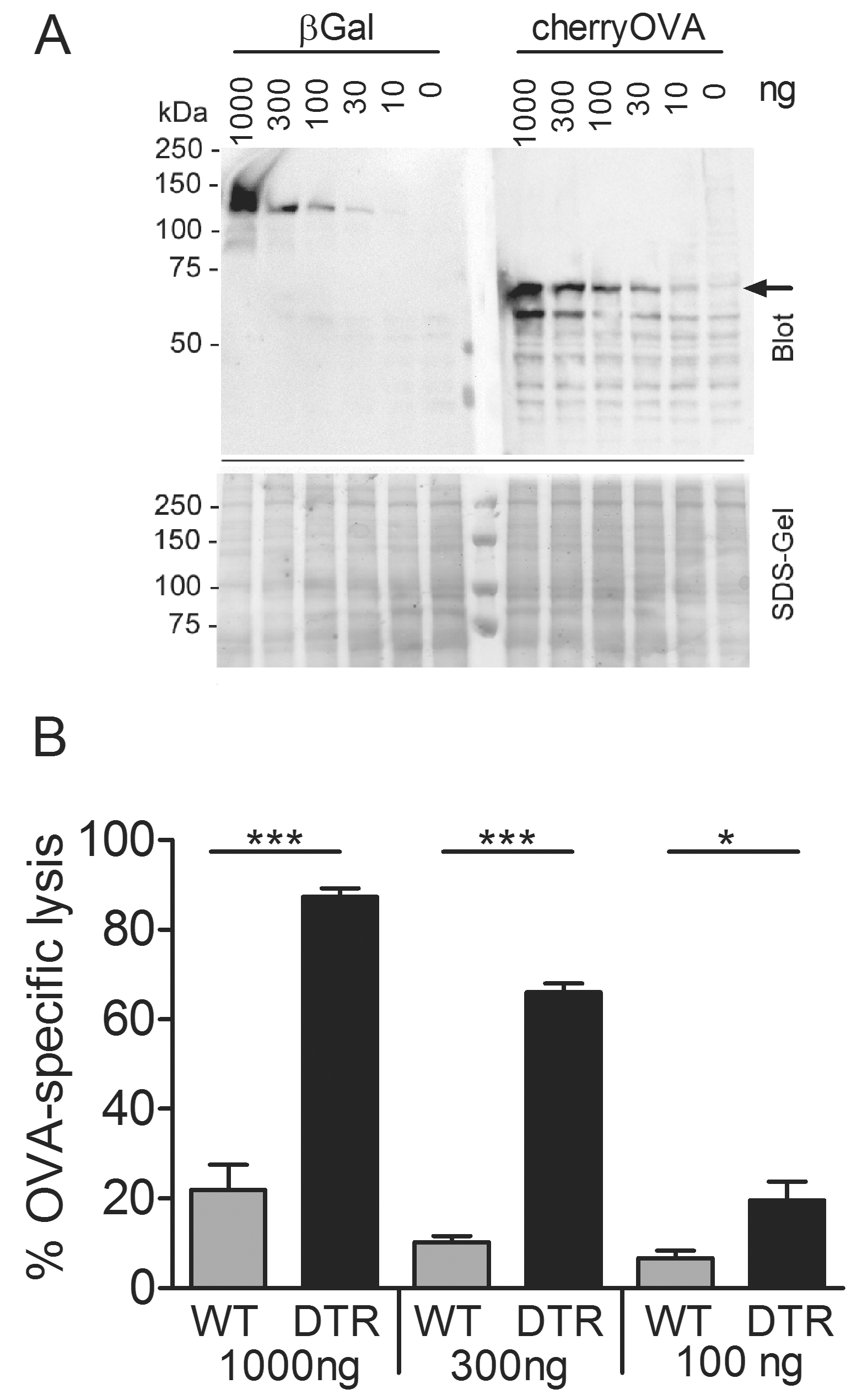

Supplement: S5 Fig — (A) Western Blot of Pam212 keratinocytes transfected with the indicated doses of pCI-βGal or pCI-cherryOVA and cultured for two days. Blots were incubated with polyclonal anti-βGal or anti-OVA antisera, respectively, followed by peroxidase-labeled secondary antibody and subsequent luminogenic development. Luminescence was recorded on ChemiDoc MP imaging system (BioRad). The uppermost band in the right panel corresponds to the full length fusion product with an expected MW of 74 kDa. The second-largest band could result from premature translation stop or degradation of the gene product; lower bands are probably unspecific signals that are equally intense in non-transfected cells. Lower Panel: SDS-gel stained with Coomassie Blue after blotting is shown as a loading control. (B) LangDTR or B6 WT mice were injected once with 1μg DT and GG-immunized 1wk later with different doses of pCI-OVA (100, 300,1000ng plasmid/GG shot). Mice were boosted with the same doses after 1 wk and analyzed 1wk later. Specific lysis of SIINFEKL-pulsed syngeneic target cells injected into GG-immunized langDTR and WT mice. Data represent means ± SD of groups of 5 mice and are representative of two experiments. (TIF) [file pone.0128722.s006.tif]
